# Anomalous dispersion in coupled surface plasmons and excitons

**DOI:** 10.1515/nanoph-2025-0036

**Published:** 2025-05-26

**Authors:** Leila Hesami, Md Golam Rabbani Chowdhury, Mikhail A. Noginov

**Affiliations:** Center for Materials Research, Norfolk State University, Norfolk, VA, 23504, USA

**Keywords:** Rhodamine laser dyes, dispersion, strong coupling, mixture dye, Kretschmann geometry

## Abstract

We studied dispersion in Rhodamine laser dyes in the Kretschmann geometry and found (i) multi-branch “staircase” dispersion curves in singly doped and double doped PMMA polymer, (ii) emergence of the new dispersion “fork” branch, (iii) unparallel dispersion and coupling in the mixture of two different dyes, and (iv) effect of high dye concentration on strong coupling without metal.

## 1 Introduction

Light–matter interaction is of great importance for both fundamental science and applications, including control of spontaneous emission [1], [2], chemiluminescence [3] energy transfer [4], [5], [6], [7], [8], [9], chemical reactions [10], [11], [12], [13], electrical resistivity [14], [15], surface potentials [16], [17], polaritonic lasers [18], [19], [20] and many others [20], [21].

Although less stable than some inorganic media, dye-doped polymers (e.g. Rh590-PMMA, Rh610-PMMA and their analogues Rh6G and RhB), are routinely used in nanophotonic research because of their strong absorption and emission [22], [23], high optical gain [24], stimulated emission [25], and coupling with surface plasmons [20], [21], cavities [26] or other molecules. Our studies of intriguing properties of dye-doped polymers in the Kretschmann geometry are reported below.

Although reflection and transmission are among the most studied strong coupling phenomena, we have observed their highly unusual dispersion behavior in single-doped (with Rh590 dye or Rh610 dye) and co-doped PMMA polymers, which, according to our knowledge, has never been reported in the literature.

**Figure 1:**
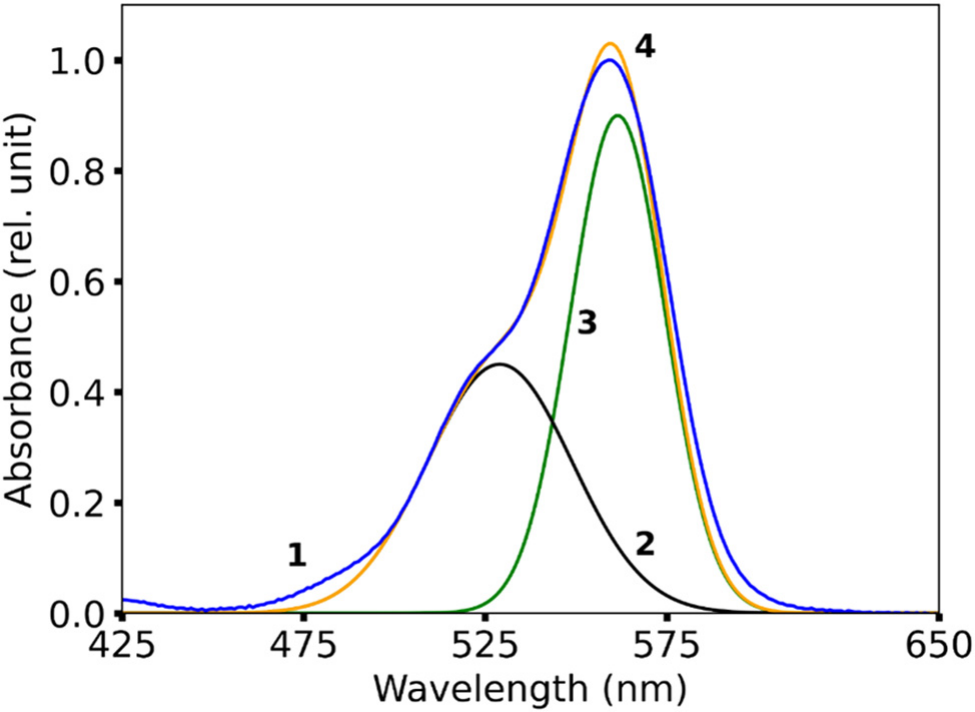
Absorption spectra of Rh610:PMMA. Trace (1) – Experimental spectrum normalized to unity; Traces (2) and (3) – Fits of the experimental spectrum with two Gaussian functions; Trace (4) – Sum of traces (2) and (3), representing the overall fit.

Following Refs. [27], [28], we fabricated a series of Kretschmann geometry samples and dye-doped PMMA films on glass, with concentrations of Rhodamine dyes (Rh590 and Rh610) in the polymer (PMMA) ranging between 0 g/L and 1,260 g/L and studied their absorption and reflection spectra (Figure 1). The angular and spectral positions of the dips in the experimental spectra resulted in the dispersion curves discussed below.

**Figure 2:**
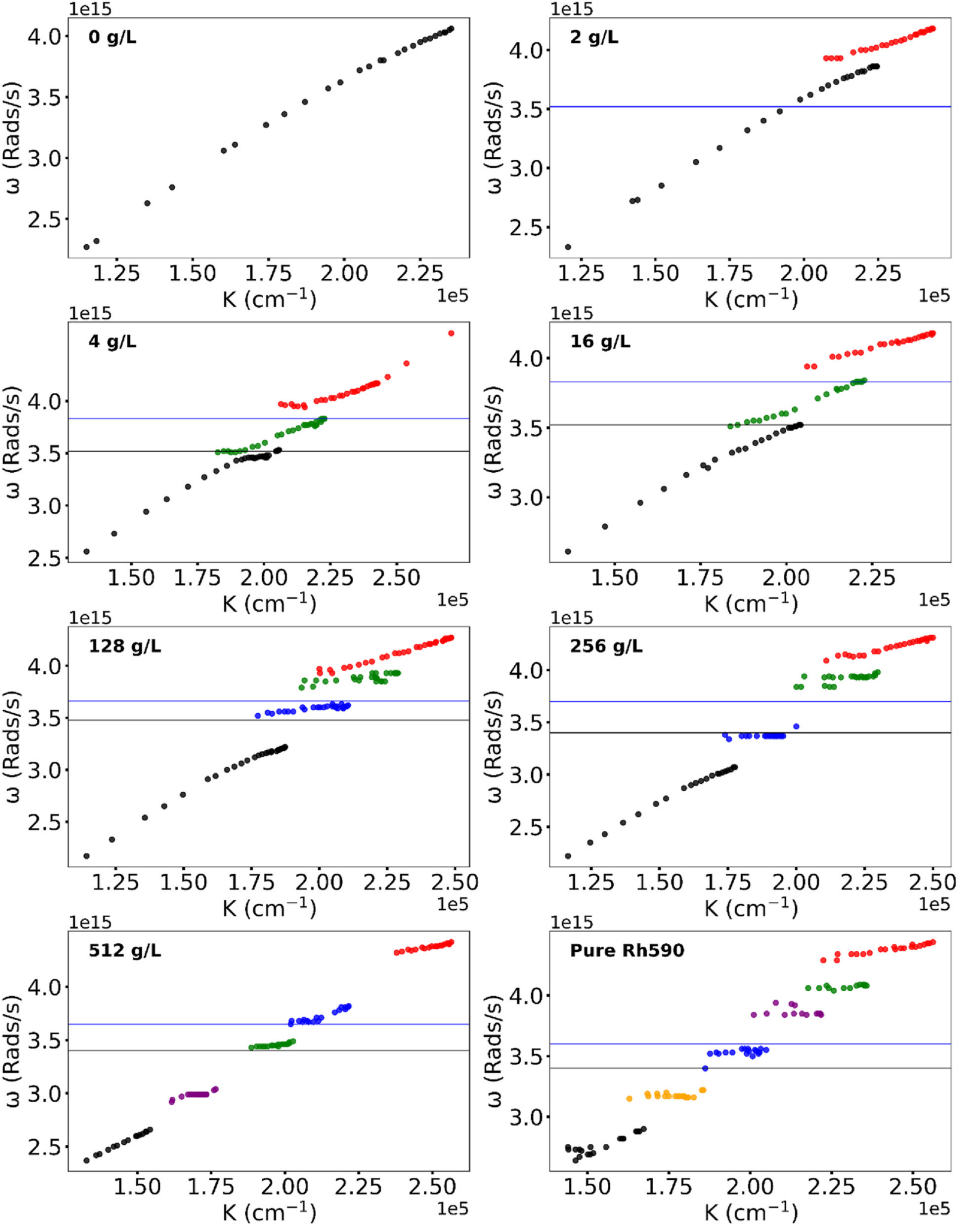
Dispersion curves in Kretschmann geometry samples at different Rh590 dye concentrations.

**Figure 3:**
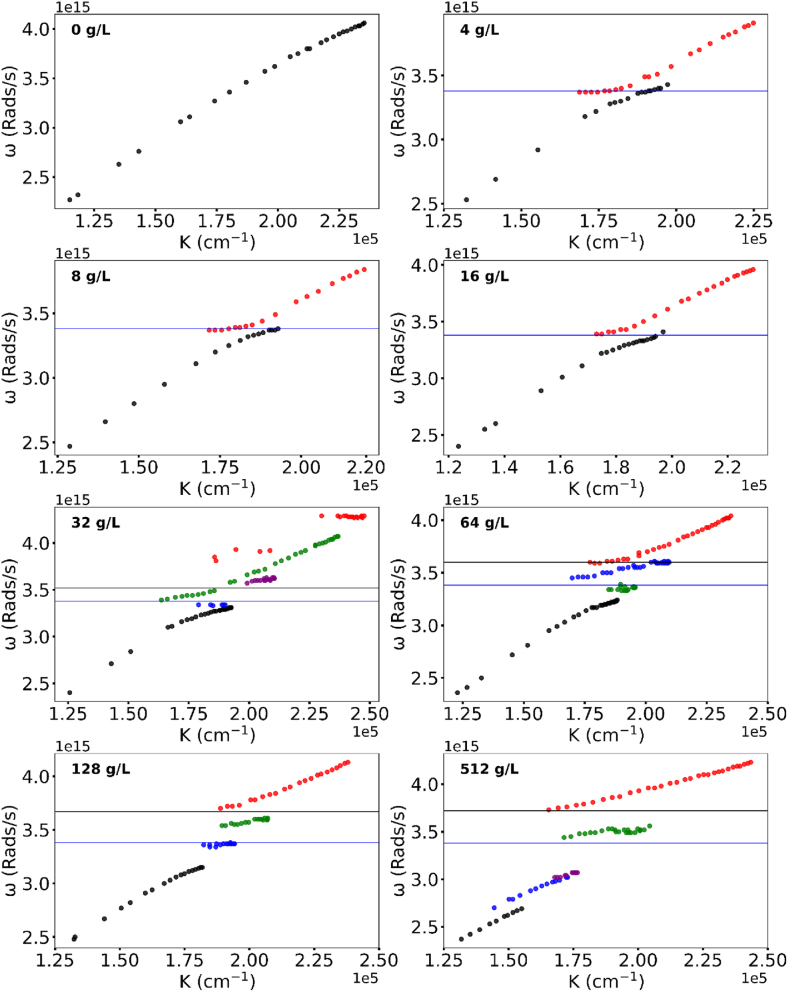
Same as Figure 2 for Rh610 dye.

**Figure 4:**
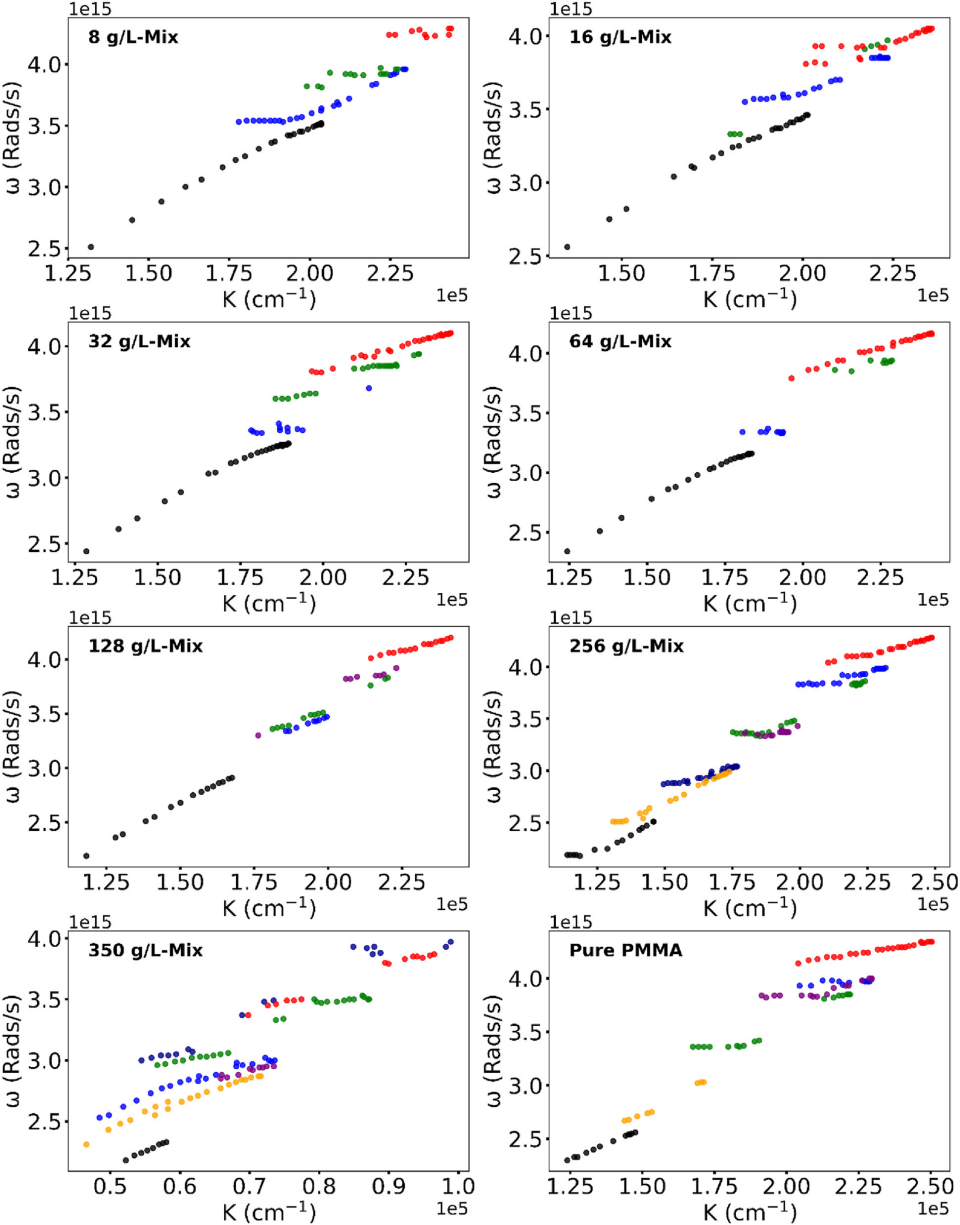
Same as Figures 2 and 3 for the mixture of Rh590 and Rh610 dyes.

**Figure 5:**
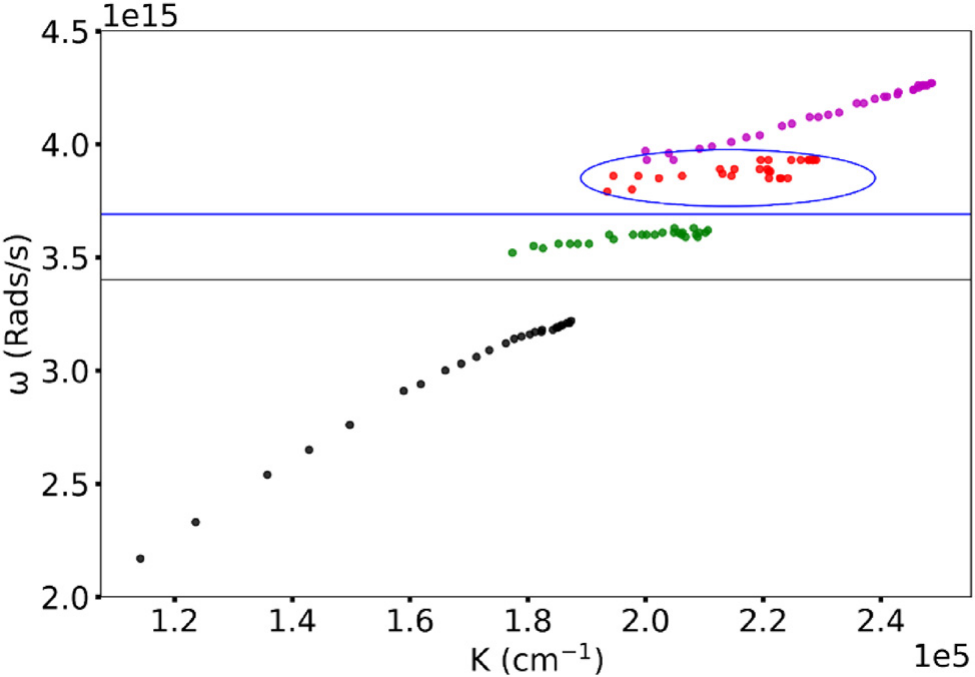
Emergence of the new dispersion branch in Rh590, at *n* = 128 g/L (encircled by an ellipse). Horizontal lines correspond to the main peak and the shoulder in the Rh590 absorption spectrum.

**Figure 6:**
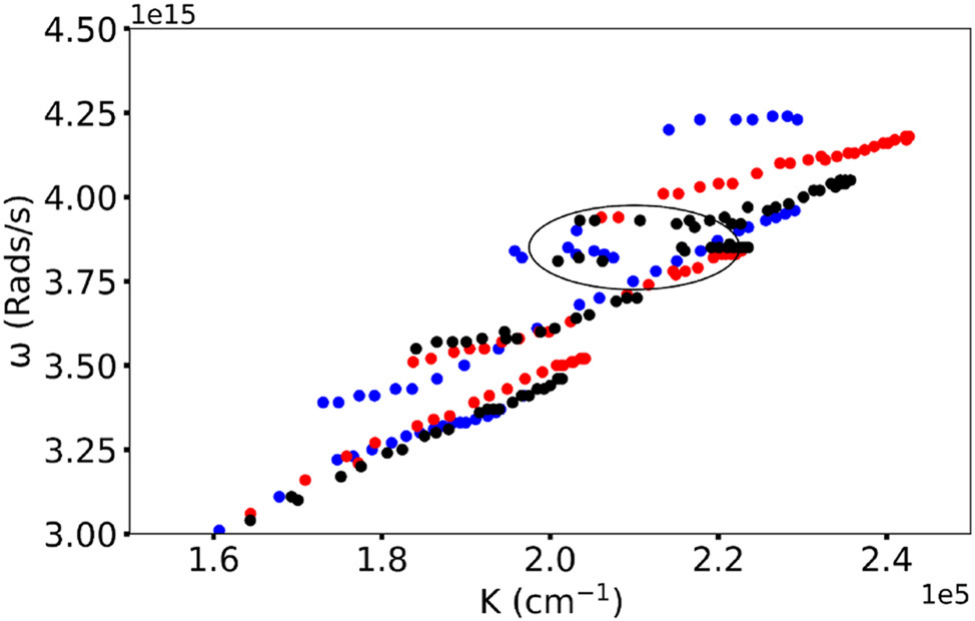
Dispersion curve of the 16 g/L Rh590 (red), Rh610 (blue), and a mixture of Rh590 and Rh610 (black). In the encircled area, the dispersion curve of the mixture is different from the sum of the components.

**Figure 7:**
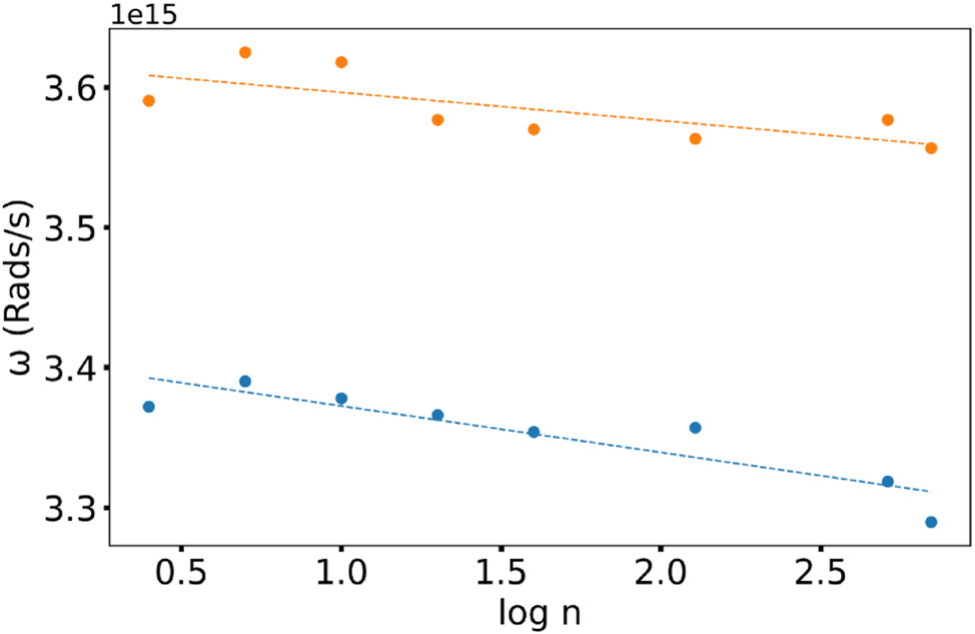
Concentration dependence of the spectral position of the main peak (blue line) and shoulder (Orange) in Rh610:PMMA.

In brief, we fabricated a variety of plasmonic and photonic samples and studied their optical properties as explained in the Supplemental information section. We have observed (i) multi-branch “staircase”-like dispersion curves of surface plasmon polaritons at high concentrations of Rh590 and Rh610 dyes (Figures 2–4), (ii) emergence of a new “fork” branch of the dispersion curve (Figure 5), (iii) dispersion in co-doped donor–acceptor system (Figure 6), and (iv) effect of dye–dye interaction on spectral positions of the absorption bands (Figure 7).

## 2 Anomalous dispersion of surface plasmons

Experimentally, in pure PMMA (no dye) the dispersion curve is formed by a single branch, in agreement with the literature [20], [29], Figures 2 and 3.

**Rh590** At modest Rh590:PMMA dye concentration (*n* = 2 g/l), the dispersion curve has two branches and one Rabi splitting, characteristic of a single absorption band, whose spectral position corresponds to the maximum of the dye’s absorption band [20], [29]. At higher Rh590 dye concentrations (*n* = 4 g/l and 16 g/l), the dispersion curve has three branches and two Rabi splits, characteristic of a peak and a shoulder of the dye absorption band [20], [29]. At *n* = 64 g/L and *n* = 128 g/l, the Rh59 dispersion curves have four branches (particularly noisy at *n* = 64 g/l) and three Rabi splittings. Intriguingly, in Rh590:PMMA at *n* = 64 g/l and *n* = 128 g/l, the upper dispersion branch features a “fork” splitting (Figure 5). This is the evidence of the emergence (or birth) of the fourth branch of the dispersion curve. In pure dyes (without PMMA), the dispersion curve in Rh590 splits into six branches (Figure 2).

**Figure 8:**
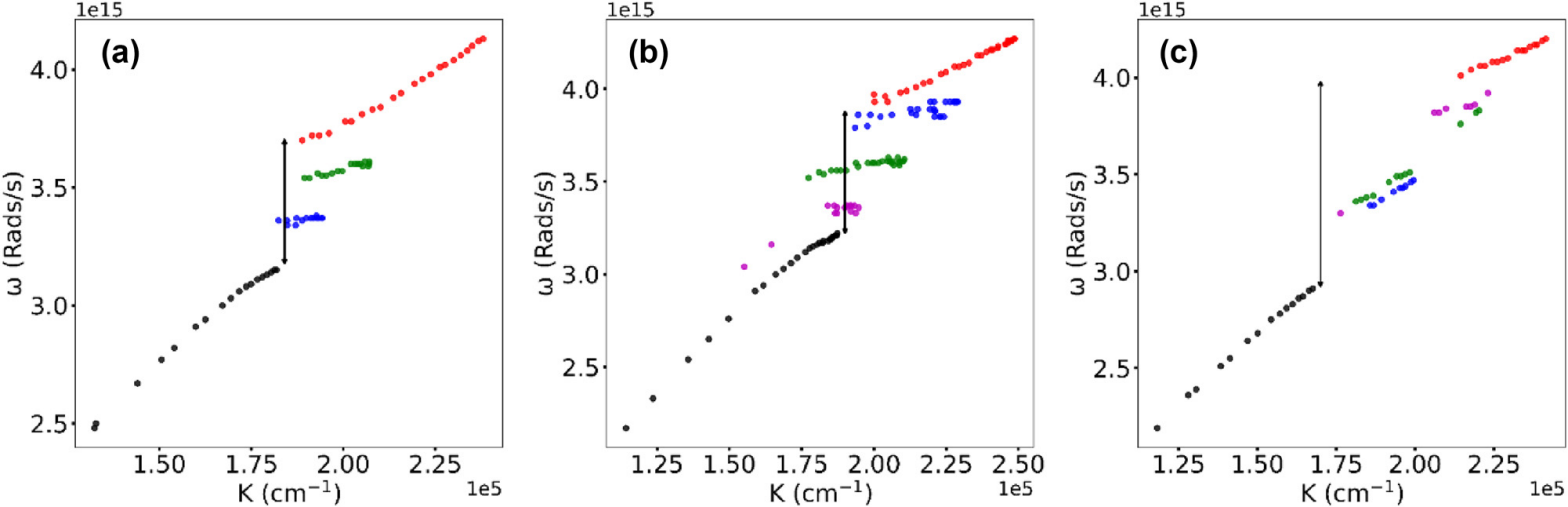
Black arrow: Energies of the lower polariton branches subtracted from the lowest energies of the upper polariton branches. (a) Rh610, (b) Rh590, (c) mix of Rh590 and Rh610. *n* = 128 g/l.

**Figure 9:**
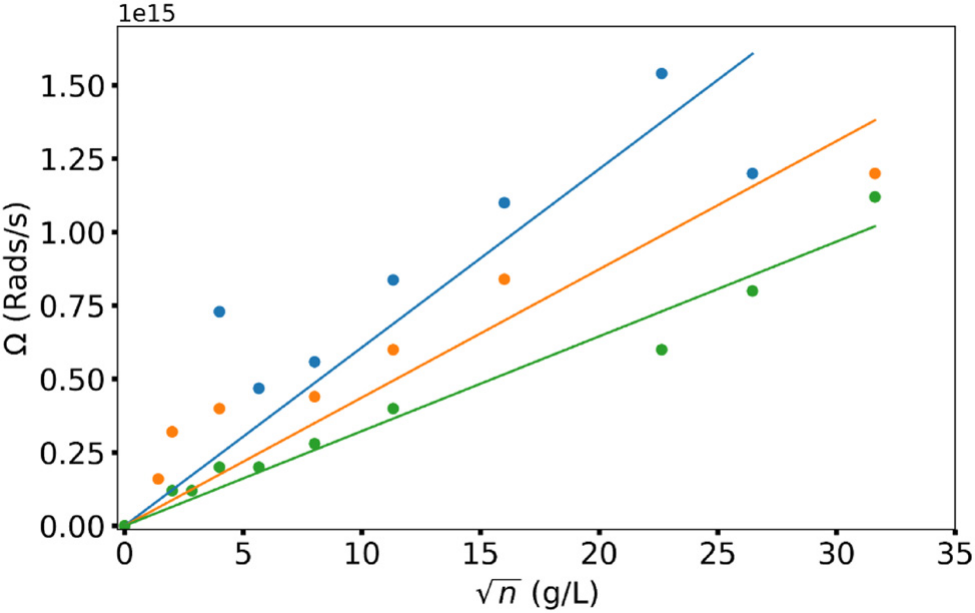
Rabi splitting proportional to the square root of the concentration. Green – Rh610, Orange – Rh590, blue – mix of Rh590 and Rh610.

**Rabi splitting** We then subtracted the highest energies of the lower polariton branches from the lowest energies of the upper polariton branches, Figure 8, and found the resultant Rabi energy to be proportional to the square root of the dye concentration [20], Figure 9. This is the theoretical prediction known for dispersion curves consisting of two branches and one Rabi splitting [20]. Note that no predicted Rabi energy scaling was observed when energy distances between *neighbo*ring branches of the dispersion curves (steps of the staircase) were measured and analyzed.

**Rh610** Qualitatively the same anomalous dispersion curves have been observed in Rh610 dye. However, the dispersion curve in Rh610 (at maximal dye concentration, no PMMA) splits only into four branches (Figure 3). Furthermore, at nominally the same Rh610 dye concentration (*n* = 16 g/l), the dispersion curve still had two branches and one Rabi splitting, similar to Rh590 dye at a lower concentration. The energy difference between the upper and lower polaritons in Rh610 had the same square root Rabi scaling as that in Rh590.

## 3 Coupling and dispersion in donor–acceptor systems

We then compared the dispersion curves in the mixture of Rh590 and Rh610 PMMA dye-doped polymers with those of the same Rh590:PMMA and Rh610:PMMA polymers taken separately and found that, as a rule, the dispersion curves in the mixtures of dyes (although noisy) can be found in the dispersion curves of individual dyes. Vice versa, the dispersion branches in the mixtures can be found in one of the dispersion curves of individual dyes. An exception from this rule is encircled in Figure 6, where one of the top branches of the mixture does not exist in Rh590:PMMA or Rh610:PMMA taken separately. Likewise, some dispersion branches of individual dyes do not exist in the dispersion curves of the mixtures. Thus, the dispersion curves of the Rh590:PMMA/Rh610:PMMA mixed samples can be more than a simple “geometrical” summation of the Rh590:PMMA or Rh610:PMMA dispersion curves, suggesting an important role played by donor–acceptor energy transfer in a strong coupling regime.

## 4 Effect of strong coupling in Rh590 and Rh610 molecules without metals

According to Ref. [22], the S0–S1 absorption band of R6G dye (very similar to Rh590) consists of a major electronic transition peak and a vibronic shoulder peak, Figure 1. Below, we study the concentration dependence of both transitions.

Experimentally, we prepared a series of Rh590:PMMA and Rh610:PMMA thin films on glass, measured their absorption spectra, and fitted them with two Gaussian functions. We found that the maximum of the major Gaussian peak is close to the maximum of the experimental absorption band. At the same time, the shoulder peak is much weaker, and its strength grows with an increase in the dye concentration, in agreement with Ref. [22]. Next, we measured the maximal spectral positions of the main peak and the shoulder and plotted them as the function of the dye concentration. We found that with increase of the dye concentration, (i) the spectral maxima of both the main peak and the shoulder are shifted to smaller frequencies and (ii) the energy splitting between the main peak and the shoulder was getting larger, Figure 7. While the latter phenomenon can be qualitatively explained in terms of the non-degenerate second-order perturbation model, the former requires thorough theoretical analysis. It tentatively can be explained by the existence of the S0–S2 absorption band at 350 nm, however, the latter band appears to be too small.

## 5 Summary

We studied dispersion in Rhodamine laser dyes in the Kretschmann geometry and found (i) multi-branch “staircase”-like dispersion curves of surface plasmon polaritons at high dye concentrations, (ii) emergence of a new “fork” branch of the dispersion curve, (iii) dispersion in co-doped donor–acceptor system, and (iv) effect of dye–dye interaction on spectral positions of the absorption bands.

## Supplementary Material

Supplementary Material Details
