# Supplementary material for: Anomalous dispersion in coupled surface plasmons and excitons
Source: Nanophotonics. 2025 May 26;14(13):2251–8. doi: 10.1515/nanoph-2025-0036 (PMC12199556; doi:10.1515/nanoph-2025-0036)
Supplement: Supplementary file 1 — Supplementary Material Details [file j_nanoph-2025-0036_suppl_001.docx]

**Supplemental Information. Methods**

**Experimental samples**

The majority of samples in our experiments were right-angle high-index Kretschmann geometry prisms with ~ 40 nm silver films deposited on their hypothenuse surfaces and dye doped polymer films deposited on the top. The samples made for transmission measurements had no silver films were deposited using the Nano 36 Thermal Evaporation Deposition System (from Kurt J Lesker, Jefferson Hills, PA, USA) and their thickness was measured with a Dek Tak XT profilometer (from Bruker).

In preparation of dye/doped PMMA films, dye and polymer were dissolved in dichloromethane (DCM) in ultrasonic bath at t=27C for 60 minutes [Dye = (Rhodamine R590, Rhodamine Rh610 or mixture of Rh 590 and Rh 610); polymer = (poly(methyl methacrylate) (PMMA)].The dye solution was drop-cast onto the silver film, creating polymer films with thicknesses of 2-3 μm.


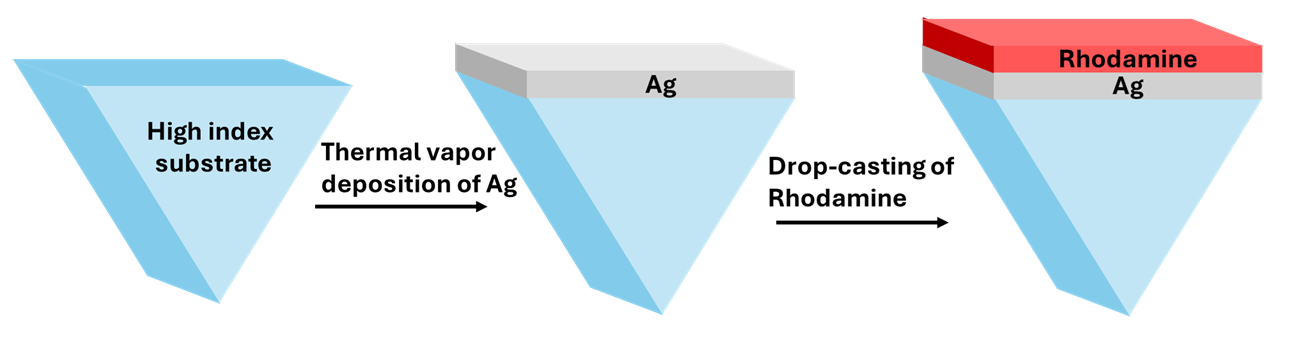


Figure 10. Chart Flow: Preparation of the Kretschmann geometry samples (high index prism/plasmonic metal/low index dielectric).

**Experiments**

In majority of experiments, Kretschmann geometry prisms were mounted onto a goniometer placed in the sample chamber of the spectrophotometer (Lambda 900 from Perkin Elmer), reconfigurable sets of right-angle prisms were used to guide the reflected light to the integrating sphere (Fig.10), and the p-polarized reflection was measured as a function of angle (Fig.11). The same spectrometer (in a conventional configuration) was used to measure transmission of dye-doped polymeric films.


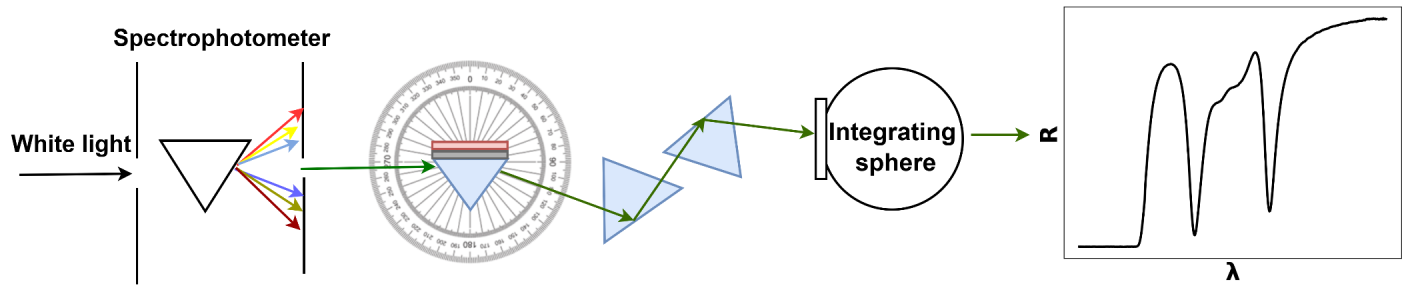


Figure 11. (Left): Schematic of the spectrophotometer set-up used to record reflectance spectra in the Kretschmann geometry. (Right): Typical reflection spectrum of the Kretschmann geometry sample.

**Data processing**

To understand the optical response of dye doped PMMA layers, several reflection spectra of the Kretschman geometry was measured at multiple angles using a rotating stage (shown in Fig. 11) yielding one or multiple dips. The intensity for each dip and the corresponding frequency (ω_measured_) was determined from these reflection spectra. This allows to calculate the dispersion relation between the wave-vectors and the Rabbi frequency (or multiple frequencies), k(ω) using the relationship ω= ω_measured_, k=sin(θ)*n, where θ is the internal incidence angle and n is the prism’s refraction angle as shown in Figs. 2,3,4. This analysis helps in characterizing the plasmonic interactions and evaluating the effect of the dye-doped PMMA layer on the resonance behavior.
